# Global sequence diversity of the lactate dehydrogenase gene in *Plasmodium falciparum*

**DOI:** 10.1186/s12936-017-2157-5

**Published:** 2018-01-09

**Authors:** Phumin Simpalipan, Sittiporn Pattaradilokrat, Pongchai Harnyuttanakorn

**Affiliations:** 1 Zoology Ph.D. Programme, Faculty of Science, Chulalongkorn University, Bangkok, 10330 Thailand; 2 Department of Biology, Faculty of Science, Chulalongkorn University, Bangkok, 10330 Thailand; 3 Veterinary Parasitology Research Group, Department of Pathology, Faculty of Veterinary Science, Chulalongkorn University, Bangkok, 10330 Thailand

**Keywords:** Genetic diversity, DNA sequencing, Lactate dehydrogenase, Rapid diagnostic tests, Malaria, Purifying selection

## Abstract

**Background:**

Antigen-detecting rapid diagnostic tests (RDTs) have been recommended by the World Health Organization for use in remote areas to improve malaria case management. Lactate dehydrogenase (LDH) of *Plasmodium falciparum* is one of the main parasite antigens employed by various commercial RDTs. It has been hypothesized that the poor detection of LDH-based RDTs is attributed in part to the sequence diversity of the gene. To test this, the present study aimed to investigate the genetic diversity of the *P. falciparum ldh* gene in Thailand and to construct the map of LDH sequence diversity in *P. falciparum* populations worldwide.

**Methods:**

The *ldh* gene was sequenced for 50 *P. falciparum* isolates in Thailand and compared with hundreds of sequences from *P. falciparum* populations worldwide. Several indices of molecular variation were calculated, including the proportion of polymorphic sites, the average nucleotide diversity index (*π*), and the haplotype diversity index (*H*). Tests of positive selection and neutrality tests were performed to determine signatures of natural selection on the gene. Mean genetic distance within and between species of *Plasmodium ldh* was analysed to infer evolutionary relationships.

**Results:**

Nucleotide sequences of *P. falciparum ldh* could be classified into 9 alleles, encoding 5 isoforms of LDH. *L1a* was the most common allelic type and was distributed in *P. falciparum* populations worldwide. *Plasmodium falciparum ldh* sequences were highly conserved, with haplotype and nucleotide diversity values of 0.203 and 0.0004, respectively. The extremely low genetic diversity was maintained by purifying selection, likely due to functional constraints. Phylogenetic analysis inferred the close genetic relationship of *P. falciparum* to malaria parasites of great apes, rather than to other human malaria parasites.

**Conclusions:**

This study revealed the global genetic variation of the *ldh* gene in *P. falciparum*, providing knowledge for improving detection of LDH-based RDTs and supporting the candidacy of LDH as a therapeutic drug target.

**Electronic supplementary material:**

The online version of this article (10.1186/s12936-017-2157-5) contains supplementary material, which is available to authorized users.

## Background

Accurate diagnosis of malaria is critical for case management and prevents unnecessary anti-malarial drug treatment. Microscopic diagnosis is considered a gold standard method for clinical diagnosis. Microscopic examinations of blood films have many limitations, including the requirement of high parasitaemia levels, long sample preparation time, and, most importantly, the required skills for diagnostic interpretation. To overcome this problem, a number of diagnostic tools that make use of conserved malaria antigens have been developed. The antigen-detecting rapid diagnostic test (RDT) is regarded as an alternative malaria diagnostic method that has been proven to be accurate, sensitive, quick and easy to interpret. RDTs detect malaria parasite specific antigens in blood through immunochromatography and can give positive or negative results for a sample at thresholds pre-set by manufacturers.

Different types of RDTs use different types of monoclonal antibodies or combinations of antibodies to detect and/or differentiate *Plasmodium* antigens. Some antibodies aim to detect a particular species. For instance, anti-histidine-rich protein 2 (HRP2) enables the detection of *Plasmodium falciparum*. On the other hand, anti-lactate dehydrogenase (LDH) is an example of a pan-malarial antibody, enabling detection of all types of human *Plasmodium* species. There are multiple formats of LDH-based detecting RDTs that are routinely employed for clinical malaria diagnostics. This includes OptiMAL-IT (Cressier, Switzerland), which identifies and differentiates *P. falciparum* from non-*P. falciparum* species (*Plasmodium vivax*, *Plasmodium ovale* and *Plasmodium malariae*) on the basis of *P. falciparum* LDH in a patient’s whole blood [1]. Several investigations have reported the performance of OptiMAL-IT assays in various endemic areas. While a general consensus has emerged that they performed well collectively, some studies have indicated that the efficacy of OptiMAL might vary from one geographical location to another [2–7]. Variation of RDT sensitivity in most studies has showed a correlation with low blood parasitaemia level, but some studies have showed variable sensitivity even in the presence of high blood parasitaemia [4, 6]. For example, the efficacy of the OptiMAL assay was evaluated in a cross-sectional survey in Thailand in 2001 [7]. The data showed that the sensitivity of the OptiMAL assay for *P. falciparum* was 25% with > 500 parasites/μl and 10.5% with > 100 parasites/μl. It is not clear from the studies whether the poor performance of RDTs was due simply to how they were engineered or whether it was due to the genetic variability of LDH itself. The analysis of polymorphism of the gene encoding HRP2 in *P. falciparum* natural isolates from different regions showed that the observed polymorphism could contribute significantly to the low sensitivities of *P. falciparum* HRP2-based RDTs [8, 9]. However, whether the genetic diversity of the gene encoding LDH of *P. falciparum* led to the poor performance of LDH-detecting RDTs has not been evaluated.

There are few publications that have investigated the genetic polymorphism of the *ldh* gene in natural *P. falciparum* populations. A study by Talman showed that there was a single haplotype of *ldh* sequences in 49 *P. falciparum* natural isolates from various geographical locations, which were identical to *P. falciparum* reference strain 3D7 [10]. Later, sequence analysis of *P. falciparum ldh* derived from *P. falciparum* populations in India, Iran and Madagascar [11–13] identified 4 novel *P. falciparum ldh* allelic types in parasite populations. Thailand is considered one of the key endemic areas for malaria and the hot spot for multi-drug resistance. To date, there is no published data of *P. falciparum ldh* genetic diversity in Thailand. To address this deficiency, the present study aimed to investigate the genetic diversity of *P. falciparum* populations in Thailand. The goals were then extended to perform systematic analysis of *P. falciparum ldh* sequences deposited in public databases. The outcome of this study enhances the understanding of the genetic diversity, genetic structure and distribution patterns of *P. falciparum ldh* in Thailand and worldwide parasite populations. This will aid in the selection of appropriated epitopes for improving RDTs. Moreover, as LDH is a promising drug target, the basis of genetic structure in this study will help to identify the proper inhibitors for the design of LDH-based anti-malarial drugs.

## Methods

### Parasite sample

A total of 50 isolates of the human malaria parasite *P. falciparum* used in the present study were maintained from the Malaria Research Laboratory, Department of Biology, Faculty of Science, Chulalongkorn University, Bangkok, Thailand. These parasites were isolated from Thai patients who were admitted to malaria clinical centres at the Thailand–Myanmar border in Tak (n = 1), Mae Hong Son (n = 10), Kanchanaburi (n = 14), and Ranong (n = 11), the Thailand–Laos border in Ubon Ratchathani (n = 4) and the Thailand–Cambodia border in Trat (n = 10) [14] (see Additional file 1). After microscopic examination, the parasites were culture-adapted using a standard candle jar method [15]. The parasite lines were genotyped with microsatellites and merozoite surface protein-3 gene for species identification [16, 17].

### Amplification and DNA sequencing of the *Plasmodium falciparum ldh* gene

Blood-stage malaria parasites were expanded in laboratories and harvested for genomic DNA preparation. The phenol/chloroform extraction method was employed, as previously described [14]. Genomic DNA was dissolved in TE buffer (pH 8.0) and stored at − 20 °C until use. PCR amplification of the *ldh* gene utilized two PCR primers with the sequences LDH-F1 5′-ATG GCA CCA AAA GCA AAA ATC GTT T-3′ and LDH-R1 5′-TTG CAT TTG TTT CTC TCT TTG TTG CA-3′, corresponding to nucleotide positions 1041824–1041850 and 1041358–1041382 of chromosome 13 from *P. falciparum* strain 3D7 (Accession Number: NC_004331), respectively. Total PCR volumes of 50 μl contained 200 μM of dNTPs, 2 mM of MgCl_2_, 0.2 μM of primers, 200–300 ng of parasite DNA and 1 unit of *Taq* polymerase enzyme in 1× *Taq* PCR buffer (Biotechrabbit, Germany). PCR amplification started with 95 °C for 1 min, followed by 35 cycles of 95 °C for 40 s, 60 °C for 40 s and 72 °C for 1.20 min, with a final extension at 72 °C for 5 min. PCR products were analysed by standard agarose gel electrophoresis. All reactions produced a single band with an estimated size of ~ 1000 bp. Subsequently, DNA sequencing was performed using BigDye™ Terminator Cycle Sequencing kit (Applied Biosystems) on an ABI3730XL DNA analyser. Sequencing primers were the PCR primers as well as two additional sequencing primers (LDH-F2 5′-CAT CAA GAT TGA AGT ATT ACA TAT CTC-3′ and LDH-R2 5′-TCT TGT AAA GGG ATA CCA CCT ACA GTA-3′) corresponding to nucleotide positions 1041936–1041962 and 1042384–1042409 of chromosome 13 from *P. falciparum* strain 3D7. Sequences of the *ldh* gene were visualized using BioEdit (version 7.2.6).

### Retrieval of *ldh* sequences from human, gorilla, chimpanzee, and simian malaria parasites

Nucleotide sequences of *P. falciparum ldh* genes from India, Iran and Madagascar were available from the published literature [11–13] (see Additional file 2). *ldh* sequences of *P. vivax* in South Korea, China, India and Iran were previously described [11, 12, 18], while the *ldh* sequence of *P. ovale* strain Harding was retrieved from a previous report by Brown et al. [19] (see Additional file 3). *ldh* sequences of malaria parasites in gorillas and chimpanzees from Cameroon and Democratic Republic of Congo were retrieved from two reports by Liu [20, 21].

Furthermore, to retrieve deposited sequences of *Plasmodium ldh* gene from the NCBI database, BLAST searches were performed using the nucleotide sequence 5′ ATG GCA CCA AAA GCA AAA ATC GTT TTA GTT 3′ as a query sequence against the nucleotide collection (nr/nt) database of all plasmodia (Taxid, 5820). Then, complete or partial sequences with an annotation “lactate dehydrogenase” from *Plasmodium* species of humans (*P. falciparum*), gorillas (*Plasmodium gaboni*), chimpanzees (*Plasmodium reichenowi*) and macaques (*Plasmodium knowlesi*) were manually selected (see Additional file 3). Homology of the putative *ldh* sequences was determined by sequence alignment.

Additional unpublished *ldh* nucleotide sequences from *Plasmodium* parasites deposited in the PlasmoDB database [22] were obtained using a key word search for “lactate dehydrogenase”. A total of 202 *P. falciparum ldh* sequences were deposited by Christopher V Plowe (Howard Hughes Medical Institute/Center for Vaccine Development, University of Maryland), Dan Neafsey (The Broad Institute), Elizabeth Winzeler (The Scripps Research Institute), Alfred Amambua-Ngwa (Medical Research Council, Fajara Banjul, The Gambia), and Dominic Kwiatkowski (Oxford/Wellcome Trust Sanger Institute) (unpublished data). The *P. knowlesi ldh* sequence was deposited by Arnab Pain [23]. The *Plasmodium cynomolgi ldh* sequence was deposited by Tachibana et al. [24]. The *P. gaboni* and *P. reichenowi ldh* sequences were deposited by Sundararaman et al. [25] and Otto et al. [26], respectively.

### Population genetic analysis of the *Plasmodium falciparum ldh* gene

*ldh* sequences from *Plasmodium* parasites were aligned and edited using the MUSCLE alignment algorithm [27] and the Alignment Editor tool in MEGA 7.0 [28]. The numbers of *ldh* alleles, non-synonymous and synonymous SNP sites, and LDH isoforms (variants of polypeptides) in each *Plasmodium* species were analysed using the BioEdit sequence editor (version 7.2.6) and DnaSP (version 5.10.1) [29]. Nucleotide diversity (*π*) was extrapolated from the number of nucleotide differences between two sequences per nucleotide site as previously described [30]. The haplotype diversity index (*H*) was calculated from a relative frequency of each haplotype and the numbers of sequences in the dataset [31]. The mean numbers of non-synonymous substitutions per non-synonymous sites (*d*_*N*_) and synonymous substitutions per synonymous sites (*d*_*S*_) were calculated using the method of Nei and Gojobori [32] with the Hasegawa–Kishino–Yano nucleotide substitution model. *d*_*N*_–*d*_*S*_ values were calculated to investigate evidence of positive selection. Significant positive values at *P* < 0.05 indicated an overabundance of non-synonymous mutation, suggesting directional selection [33, 34].

Departures from the predictions of the neutral mode of molecular evolution were determined using Tajima’s *D*, Fu and Li’s D* and Fu and Li’s F* indices, implemented in DnaSP 5.10.1 software [35, 36]. The results of the neutrality tests were deemed to be statistically significant if the *P* value was less than 0.05 (*P* < 0.05). In addition, sliding window plots, with a window length of 90 bases and a step size of 3 bp, were generated for *d*_*N*_–*d*_*S*_ and neutrality test analyses to identify regions of *ldh* where a significant departure from neutrality was observed (*P* < 0.05). Divergence in the distributions of *ldh* alleles of *P. falciparum* populations in Africa, Asia and South America was tested for using Wright’s fixation index (*F*_*st*_) with Arlequin version 3.5 [37]. Significant differences of genetic variance for any parasite population pairs at *P* < 0.05 indicated population differentiation.

### Phylogenetic analysis

Unique *ldh* haplotypes of the malaria parasites from humans (*P. falciparum*, *P. malariae*, *P. ovale*, *P. vivax*), gorillas (*Plasmodium praefalciparum*, *Plasmodium alderi*, *Plasmodium billcollinsi*), chimpanzees (*P. reichenowi*, *Plasmodium blacklocki*, *P. gaboni*), and macaques (*P. knowlesi*, *P. cynomolgi*) were selected from the putative *ldh* sequences in the NCBI and PlasmoDB databases. Sixty-one *ldh* sequences were included in the phylogenetic tree reconstruction (Additional files 2, 3). The best-fit substitution model for the multiple sequence alignment generated by MUSCLE was determined using the Bayesian Information Criteria (BIC) strategy in jModelTest [38]. Evolutionary relationships of the aligned sequences were determined using neighbour-joining (NJ) and maximum likelihood (ML) approaches in MEGA 7.0 [28] based on the p-distance method and general time reversible substitution model with a gamma distributed shape parameter (GTR+G), respectively. The robustness of the tree topology was tested with 1000 bootstrap replicates.

## Results

### Nucleotide sequences of the *ldh* gene in Thai *Plasmodium falciparum* isolates

The *ldh* gene of *P. falciparum* was amplified from genomic DNA of 50 *P. falciparum* isolates and sequenced using a Sanger sequencing method as described in “Methods”. An analysis of the 918-bp partial sequences of the *ldh* gene, corresponding to nucleotide positions 31–948 in *P. falciparum* strain 3D7, revealed a single allelic type of *ldh*, named *L1a* (Table 1 and Additional file 1). The *L1a* allele was also identical to the sequence of *P. falciparum* strain 3D7.

**Table 1:** Single nucleotide polymorphisms (SNPs) in the gene encoding lactate dehydrogenase (LDH) of *Plasmodium falciparum*

Allele	Isoform	Nucleotide position (corresponding amino acid position)														
		36 (12)	73 (25)	85 (29)	259 (87)	399 (133)	407 (136)	450 (150)	451 (151)	513 (171)	560 (187)	563 (188)	783 (261)	814 (272)	858 (286)	891 (297)
*L1a*	LDH-1	TCA *S*	CAG **Q**	GGA **G**	GGA **G**	GTA *V*	TTA **L**	TTA *L*	GGT **G**	CCA *P*	GTT **V**	CTT **L**	TCA *S*	GAT **D**	GTT *V*	GAG *E*
*L1b*		TCG *S*	–	–	–	–	–	–	–	–	–	–	–	–	–	GAA *E*
*L1c*		–	–	–	–	–	–	–	–	CCC *P*	–	–	–	–	GTC *V*	–
*L1d*		–	–	–	–	–	–	TTG *L*	–	–	–	–	–	–	–	–
*L1e*		–	–	–	–	–	–	–	–	–	–	–	TCG *S*	–	–	–
*L2*	LDH-2	–	–	–	–	–	–	–	–	–	–	–	–	AAT **N**	–	–
*L3*	LDH-3	–	AAG **K**	–	–	–	–	–	–	–	–	–	–	–	–	–
*L4*	LDH-4	–	–	CGA **R**	CGA **R**	GTC *V*	–	–	CGT **R**	–	GGT **G**	–	–	–	–	–
*L5*	LDH-5	–	–	–	–	–	TCA **S**	–	–	–	–	CCT **P**	–	–	–	–

Positions of polymorphic nucleotides (underlined letter) in the *ldh* gene are numbered according to the *ldh* sequence of *P. falciparum* reference strain 3D7. Amino acid positions of LDH are shown in parentheses (bold, non-synonymous amino acid substitution; italic, synonymous substitution). A nucleotide substitution at position 15 (GCA/GCG) reported in *P. falciparum* Mzr-1 isolate from India (NCBI sequence ID: JN547219, [11]) was excluded in the present study. Dashes (–) indicate the nucleotide sequence of the *L1a* allele

### Nucleotide sequences of the *ldh* gene in worldwide *Plasmodium falciparum* isolates

Having demonstrated the fixation of the *ldh* allele in Thai *P. falciparum* populations, the global diversity of the *ldh* gene in *P. falciparum* was further investigated. Nucleotide sequences of the *ldh* gene in *P. falciparum* from 23 countries worldwide were retrieved from the PlasmoDB and NCBI databases and the literature (Table 2 and Additional file 2), thereby generating a database of *ldh* sequences containing 268 worldwide *P. falciparum* isolates. A comparison of the 918-bp nucleotide sequences revealed 15 single nucleotide polymorphism (SNP) sites, revealing 9 *ldh* gene alleles with a haplotype diversity index (*H*) of 0.203, an average pairwise nucleotide difference between sequences (*k*) of 0.355 and an average nucleotide diversity at each locus (*π*) of 0.0004.

**Table 2:** Geographical distribution of *ldh* alleles in natural isolates of *Plasmodium falciparum*

Origin of *P. falciparum*	*L1a*	*L1b*	*L1c*	*L1d*	*L1e*	*L2*	*L3*	*L4*	*L5*
			LDH-1			LDH-2	LDH-3	LDH-4	LDH-5
Cambodia^c^	1	0	0	0	0	0	0	0	0
P. R. China^c^	0	0	0	1	0	1	0	1	1
Indonesia^a^	0 (133)^d^	0	0	0	0	0	0	0	0
India^a^	2 (46)^d^	0	1	0	0	0	0	0	0
Iran^a^	6	1	0	0	0	0	0	0	0
Laos^c^	2	0	0	0	0	0	0	0	0
Malaysia^c^	1	0	0	0	0	0	0	0	0
The Philippines^c^	1	0	0	0	0	0	0	0	0
Thailand^b^	53	0	0	0	0	0	0	0	0
Vietnam^c^	3	0	0	0	0	0	0	0	0
The Gambia^c^	54	0	0	0	0	11	0	0	0
Ghana^c^	0	0	0	0	0	1	0	0	0
Madagascar^a^	0 (126)^d^	0	0	0	0	1	1	0	0
Mali^c^	14	0	0	0	1	8	0	0	0
Mozambique^c^	1	0	0	0	0	0	0	0	0
Senegal^c^	54	0	0	0	0	8	0	0	0
Tanzania^c^	1	0	0	0	0	0	0	0	0
Uganda^c^	7	0	0	0	0	1	0	0	0
Brazil^c^	4	0	0	0	0	0	0	0	0
Columbia^c^	1	0	0	0	0	0	0	0	0
El Salvador^c^	1	0	0	0	0	0	0	0	0
French Guiana^c^	22	0	0	0	0	0	0	0	0
Honduras^c^	2	0	0	0	0	0	0	0	0
Total	230	1	1	1	1	31	1	1	1

The numbers of nucleotide sequences from the PlasmoDB and NCBI databases are shown

^a^Published sequences of *P. falciparum ldh* in Indonesia, Madagascar, India and Iran were taken from the literature [11–13, 39]

^b^Sequence data of 50 *P. falciparum* isolates in the present study and 3 sequences of *P. falciparum* strain K1 submitted to the NCBI database

^c^Unpublished nucleotide sequences of the *ldh* gene were retrieved from the PlasmoDB and NCBI databases and used to deduce the amino acid sequences (see Additional file 2)

^d^Numbers in brackets indicate *ldh* sequences of *P. falciparum* that were identical to *L1a* of *P. falciparum* 3D7 which were reported in literature [11–13, 39], but without the NCBI sequence IDs

In Table 1, 7 SNPs detected at positions 36 (TCA/TCG), 399 (GTA/GTC), 450 (TTA/TTG), 513 (CCA/CCC), 783 (TCA/TCG), 858 (GTT/GTC) and 891 (GAG/GAA) resulted in synonymous mutations at amino acid residues 12 (S), 133 (V), 150 (L), 171 (P), 261 (S), 286 (V) and 297 (E), respectively. The nucleotide substitution frequencies at positions 12, 133, 150, 171, 261, 286, and 297 were A/G (99.8%/0.2%), A/C (99.8%/0.2%), A/G (99.8%/0.2%), A/C (99.8%/0.2%), A/G (99.8%/0.2%), T/C (99.8%/0.2%) and G/A (99.8%/0.2%). The other 8 SNPs were detected at nucleotide positions 73 (CAG/AAG), 85 (GGA/CGA), 259 (GGA/CGA), 407 (TTA/TCA), 451 (GGT/CGT), 560 (GTT/GGT), 563 (CTT/CCT) and 814 (GAT/AAT), resulting in non-synonymous amino acid substitutions at residues 25 (Q/K), 29 (G/R), 87 (G/R), 136 (L/S), 151 (G/R), 187 (V/G), 188 (L/P) and 272 (D/N), respectively. The nucleotide substitution frequencies at positions 73, 85, 259, 407, 451, 560, 563 and 814 were C/A (99.8%/0.2%), G/C (99.8%/0.2%), G/C (99.8%/0.2%), T/C (99.8%/0.2%), G/C (99.8%/0.2%), T/G (99.8%/0.2%), T/C (99.8%/0.2%), and G/A (91.1%/8.9%), respectively.

Of the 7 synonymous SNP sites, 2 SNPs at positions 36 and 891 were unique to *L1b*, whereas SNPs at positions 513 and 858 were unique to *L1c* (Table 1). The other 2 SNPs at positions 450 and 783 were unique to *L1d* and *L1e*, respectively. *L2*, *L3*, *L4* and *L5* alleles of the *ldh* gene were characterized by 8 non-synonymous SNPs. Two SNPs at positions 814 and 73 were unique to the *L2* and *L3* alleles, respectively. Four SNP sites at positions 85, 259, 451 and 560 were specific to the *L4* allele, while 2 SNP sites at positions 407 and 563 were specific to the *L5* allele.

### Signature of negative purifying selection on *ldh*

**Fig. 1:**
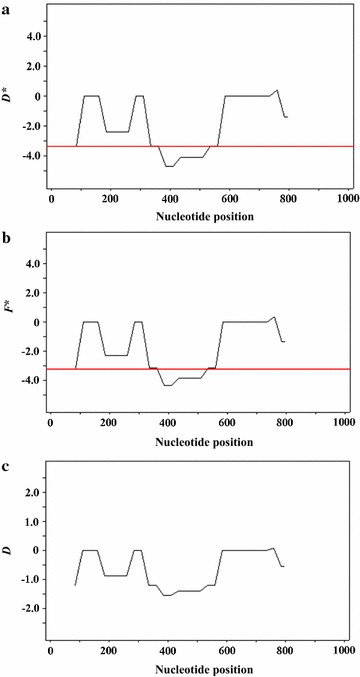
Sliding window plots of Tajima’s *D* values, Fu and Li’s D* values and Fu and Li’s F* value of *Plasmodium falciparum* lactate dehydrogenase. The plots of Tajima’s *D* values (**a**), Fu and Li’s D* values (**b**) and Fu and Li’s F* values (**c**) were analysed using default parameters, with a window length of 100 bp and step size of 25 bp. Significantly negative D* and F* statistics were detected at nucleotide positions 333–558 (corresponding to amino acid residues 111–186), suggesting a signature of negative (purifying) selection. The red line indicates *P* values < 0.05

**Fig. 2:**
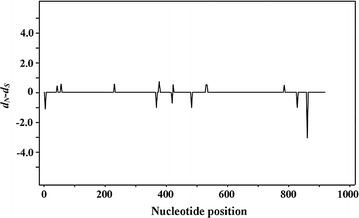
*d*_*N*_–*d*_*S*_ plot of the gene encoding lactate dehydrogenase of *P. falciparum*. Sequences used in the analysis corresponded to nucleotide positions 31–948 with respect to *P. falciparum* reference strain 3D7. The *d*_*N*_–*d*_*S*_ values of zero, equivalent to a *d*_*N*_/*d*_*S*_ ratio of 1, indicate neutral selection on the gene

Because of the observed low levels of polymorphism, the next goal was to determine signatures of purifying selection on this gene. Three neutrality tests revealed significantly negative Tajima’s *D*, Fu and Li’s D* and Fu and Li’s F* statistics of − 1.967 (*P* < 0.05), − 7.065 (*P* < 0.02) and − 6.196 (*P* < 0.02), respectively. These results indicated that the *ldh* gene of *P. falciparum* was under negative purifying selection. To determine whether specific region(s) of the *ldh* region were under selection, a sliding window plot analysis was performed, with a window of 90 bp and a step size of 3 bp. Significantly negative D* and F* statistics were detected at nucleotide positions 333–558 (Fig. 1). A sliding window plot of *D* statistics also showed negative values (0 to − 1.802), although the values did not show a significant departure from zero (Fig. 1c). Examination of the patterns of synonymous and non-synonymous substitutions was also indicative of purifying selection, which is not surprising given the low levels of observed polymorphism and the results from the Fu and Li’s D* and F* tests. The average values of the numbers of non-synonymous substitution per non-synonymous site (*d*_*N*_) and number of synonymous substitution per synonymous site (*d*_*S*_) were 0.02763 ± 0.2040 and 0.0138 ± 0.0854, respectively. The *d*_*N*_–*d*_*S*_ values of the *ldh* gene did not show a significant departure from zero (Fig. 2). Taken together, these results supported the view that the genetic conservation in the *ldh* gene of *P. falciparum* was mainly attributed to purifying selection.

### Distribution of *ldh* alleles in *Plasmodium falciparum* populations

The 918-nucleotide fragments of the *ldh* gene were translated to 306 amino acid residues, corresponding to amino acid positions 11–316 in *P. falciparum* strain 3D7. An analysis of amino acid sequences revealed 5 distinct isoforms of LDH (Table 1). Five alleles, named *L1a*, *L1b*, *L1c*, *L1d*, and *L1e*, produced the same amino acid sequence, corresponding to the LDH-1 isoform. The other 4 allelic types, *L2*, *L3*, *L4* and *L5*, encoded different LDH amino acid sequences and were designated the LDH-2, LDH-3, LDH-4 and LDH-5 isoforms, respectively. Different alleles of *ldh* showed different geographical distributions. Table 2 shows that *L1a* was the most abundant allele, and was distributed across almost all *P. falciparum* populations. *L1b*, *L1c*, and *L1d* were detected in *P. falciparum* populations in Iran, India and China, while *L1e* was present in Mali. The second most abundant allele was *L2*, which was reported in Iran, The Gambia, Madagascar, and Uganda. In contrast, 3 alleles, *L3*, *L4* and *L5*, were rare *ldh* alleles that were detected in single parasite isolates. *L3* was reported only in Madagascar, while the *L4* and *L5* isoforms were found in *P. falciparum* populations in China.

The distribution of different LDH isoforms also reflected different levels of genetic diversity of *ldh* in *P. falciparum* populations. The results showed that genetic diversity of the *ldh* gene was lowest in South America, where one isoform of LDH was detected in all *P. falciparum* populations. In contrast, *P. falciparum* populations in Iran and many African countries, including the Gambia, Ghana, Senegal, and Uganda carried the 2 most abundant isoforms of LDH (LDH-1 and LDH-2). Madagascar was the only endemic area in which 3 isoforms (LDH-1, LDH-2, LDH-3) were detected.

To further determine whether parasite populations in Asia, Africa and South America are genetically isolated, pairwise inter-population comparisons were performed for each parasite population using Wright’s fixation index (*F*_*st*_). Table 3 shows that the *F*_*st*_ values from the pairs of *P. falciparum* populations between South America and Asia and between South America and Africa were low and non-significant. The analysis indicated that the *P. falciparum* population in South America was genetically similar to *P. falciparum* populations in Africa and Asia. In contrast, a significant *F*_*st*_ value was detected between parasite populations from Africa and Asia (*P* < 0.05), suggesting genetic differentiation between these parasite populations.

**Table 3:** Pairwise *F*_*st*_ values of *ldh* haplotypes between *Plasmodium falciparum* populations in Asia, Africa and South America

	South America	Africa
Africa	0.00167 (0.12613 ± 0.0309)	–
Asia	− 0.01592 (0.99099 ± 0.0030)	0.01569* (0.00000 ± 0.0000)

Asterisk (*) indicates significant *F*_*st*_ value

### Evolutionary relationships of *Plasmodium* spp. *ldh* genes in mammals

A final goal of the present study was to compare levels of genetic diversity of *ldh* sequences in different *Plasmodium* species infecting humans and non-human primates. Nucleotide sequences of *ldh* genes of the human malaria parasites *P. vivax*, *P. ovale* and *P. malariae* were retrieved from the NCBI database, as described in “Methods”. The 49 *ldh* sequences of *P. vivax* contained 21 non-synonymous SNPs and 10 synonymous SNPs, which could be classified into 11 alleles, while the 3 *ldh* sequences of *P. ovale* contained 5 non-synonymous SNPs and 27 synonymous SNPs classified into 3 alleles (Table 4 and Additional file 3). There was only one *ldh* sequence from *P. malariae*. The level of *ldh* nucleotide diversity (*π*) in *P. ovale* (*π* = 0.0208) was greater than those of *P. falciparum* (*π* = 0.0004) and *P. vivax* (*π* = 0.0021), although the number of available sequences was much lower. The non-synonymous mutations were outnumbered by synonymous mutations in *P. ovale ldh* sequences, which again suggest that the *P. ovale ldh* gene is under strong purifying selection. In addition to the *ldh* sequences of the human malaria parasites, *ldh* sequences from parasites of non-human primates were obtained from the NCBI database, including 9 *ldh* sequences from 3 species of malaria parasites in the gorilla: *P. praefalciparum* (n = 4), *P. alderi* (n = 4) and *P. billcollinsi* (n = 1), 29 *ldh* sequences from 3 species of malaria parasites in chimpanzees: *P. reichenowi* (n = 11), *P. gaboni* (n = 15) and *P. blacklocki* (n = 3), and 2 *ldh* sequences from 2 species of malaria parasites in macaques: *P. cynomolgi* and *P. knowlesi* (Additional file 3). An analysis of non-human primate malaria *ldh* sequences showed that the highest numbers of *ldh* alleles were detected in malaria parasites isolated from chimpanzees, *P. gaboni* and *P. reichenowi*. There were, however, no differences in the levels of the nucleotide diversity of *ldh* in *P. gaboni* and *P. reichenowi* compared to other non-human primate malaria parasites, including *P. praefalciparum*, *P. alderi* and *P. blacklocki*. It should also be noted that the levels of nucleotide diversity of *ldh* genes in the non-human malaria parasites were much higher than that of *P. falciparum* but were similar to that of *P. vivax*. These data demonstrate, therefore, the different levels of genetic diversity in *ldh* genes across malaria parasite species of humans and non-human primates.

**Table 4:** Genetic diversity of the gene encoding lactate dehydrogenase in malaria parasites of humans and non-human primates

Species	Host	n	Size (bp)	nsSNP	sSNP	Allele	Isoform	*π*
*P. falciparum*	*Homo sapiens*	268	918	8	7	9	5	0.0004
*P. vivax* ^*a*^	*Homo sapiens*	49	931	21	10	11	9	0.0021
*P. ovale* ^*b*^	*Homo sapiens*	3	951	5	27	3	3	0.0208
*P. malariae* ^*d*^	*Homo sapiens*	1	898	ND	ND	1	1	ND
*P. praefalciparum* ^*c*^	*Gorilla gorilla gorilla*	4	821	1	1	3	2	0.0013
*P. alderi* ^*c*^	*Gorilla gorilla gorilla*	4	770	0	3	4	1	0.0022
*P. billcolinsi* ^*c*^	*Gorilla gorilla gorilla*	1	770	ND	ND	1	1	ND
*P. reichenowi* ^*c*^	*Pan troglodytes*	11	822	0	9	10	1	0.0025
*P. gaboni* ^*c*^	*Pan troglodytes*	15	770	1	9	14	3	0.0030
*P. blacklocki* ^*c*^	*Pan troglodytes*	3	770	1	1	3	2	0.0017
*P. cynomolgi* ^*d*^	*Macaca fascicularis*	1	770	ND	ND	1	1	ND
*P. knowlesi* ^*d*^	*Macaca fascicularis*	1	770	ND	ND	1	1	ND

*n* number of nucleotide sequences, *nsSNP* non-synonymous SNsP, *sSNP* synonymous SNPs, *ND* not determined

^a^*ldh* sequences of *P. vivax* in South Korea, China, India and Iran were taken from the literature [11, 12, 18]

^b^*ldh* sequence of *P. ovale* strain Harding derived from Brown et al. [19]

^c^*ldh* sequences from gorilla and chimpanzee malaria parasites in Cameroon and Democratic Republic of Congo were taken from Liu et al. [20, 21]

^d^Unpublished sequences deposited in the PlasmoDB database (see Additional file 3 for sequence ID)

**Fig. 3:**
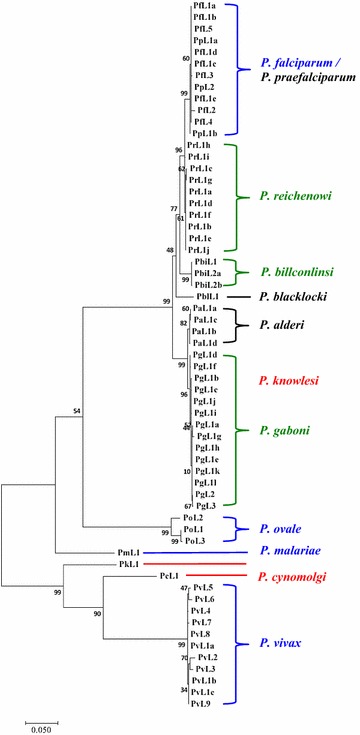
Maximum likelihood tree of 61 unique alleles of the gene encoding lactate dehydrogenase (*ldh*) from 12 *Plasmodium* parasite species. The sequences are named according to parasite species and allelic type. The first two/three letters indicate parasite species: *Pf* (*P. falciparum*), *Pm* (*P. malariae*), *Po* (*P. ovale*), *Pv* (*P. vivax*), *Pp* (*P. praefalciparum*), *Pr* (*P. reichenowi*), *Pbi* (*P. billcollinsi*), *Pbl* (*P. blacklocki*), *Pa* (*P. alderi*), *Pg* (*P. gaboni*), *Pk* (*P. knowlesi*) and *Pc* (*P. cynomolgi*). Species showed on the right side are labelled with colour representing parasite host: *Homo sapiens* (blue), *Gorilla gorilla* (black), *Pan troglodytes* (green), and *Macaca fascicularis* (red). The tree was constructed using the aligned sequences of 768 nucleotides, corresponding to nucleotide positions 52–819 in *P. falciparum* strain 3D7. Bootstrap values are shown next to the nodes. The scale bar represents nucleotide substitutions per site

Phylogenetic trees of 61 *ldh* gene alleles from the 12 species of *Plasmodium* species in human and non-human primates were constructed using the neighbour joining and maximum likelihood methods. As shown in Fig. 3, the phylogenetic tree from the maximum likelihood method yielded 2 branches of *ldh* genes. The same tree topology was generated using neighbour-joining method (Additional file 4). The major branch is represented by the sequences of the 3 human malaria parasites, *P. falciparum*, *P. malariae* and *P. ovale*, as well as the gorilla and chimpanzee *Plasmodium* sequences. The tree also inferred that the *P. falciparum ldh* gene clustered closely together with the gorilla and chimpanzee malaria parasites. It should be noted that *P. ovale* and *P. malariae* formed a sister branch to the *P. falciparum* lineage, but the sequences were more diverse and distantly related to *P. falciparum*. Interesting, the minor branch of the *ldh* gene tree was represented by the sequences of *P. vivax ldh* and the malaria parasites in macaques, *P. knowlesi* and *P. cynomolgi*. These results imply that the malaria parasites in humans may originate from different sister species of non-human primate malaria.

## Discussion

LDH is considered a major target for therapeutic drugs and for the development of RDTs. The sequence analysis of *ldh* genes for human and non-human malaria parasites has been a subject of intensive research. However, there is limited sequence information available for the *ldh* gene, especially for *P. falciparum* populations in Thailand, the hotspot of multidrug resistance. Thus, the primary goal of the present study was to investigate the genetic diversity of the *LDH* gene in natural isolates of *P. falciparum* collected from 5 localities near the Thailand–Myanmar, Thailand–Laos and Thailand–Cambodia borders. The main finding was that Thai *P. falciparum* isolates all possessed identical sequences for the *ldh g*ene, named *L1a*. Similar genotyping results of the *P. falciparum ldh* gene were found in Indonesia, where all *P. falciparum* isolates carried the *L1a* allele [39]. This suggested the fixation of the *ldh* gene in *P. falciparum* populations in Thailand and Indonesia. The findings were contrary to previous analyses of *ldh* sequences in *P. falciparum* in India, Iran and Madagascar [11–13], in which multiple *ldh* alleles (*L1b*, *L1c*, *L2* and *L3*) were reported.

To further investigate the global diversity of *ldh*, the deposited *ldh* sequences of *P. falciparum* from various geographical origins in the NCBI and PlasmoDB databases were analysed. Interestingly, the analysis identified 4 unreported alleles of *P. falciparum ldh*. The *L1d* and *L1e* alleles differed from *L1a* by 1 synonymous mutation and were found in *P. falciparum* isolates in China and Mali, respectively. *L4* and *L5* were detected in a *P. falciparum* isolate, FCC1/HN, maintained at the Institute of Tropical Medicine, First Military Medical University, Guangdong, China. Because *L4* and *L5* were found in laboratory isolates, further investigation will be required to determine whether the mutations occur in natural parasite populations or arose through technical errors. Overall, there were at least 9 alleles of the *ldh* gene in *P. falciparum* worldwide, and the *L1a* allele was the most common, circulating in all endemic regions in Asia, Africa and South America.

**Fig. 4:**
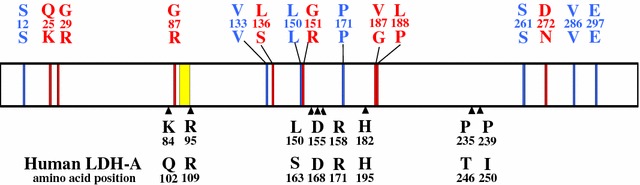
Amino acid substitution sites on *P. falciparum* LDH in comparison with human LDH. Non-synonymous substitution positions (red) and synonymous substitution positions (blue) were marked on the putative LDH of *P. falciparum* 3D7 strain (amino acid residues 1–317). The yellow block represents the location of *Plasmodium*-specific extended amino acids: DKEWN (amino acid positions 90–94). Arrowheads indicate conserved catalytic and cofactor-binding amino acid residues (black) found in *P. falciparum* LDH. Corresponding amino acid residues in human LDH-A are shown in the bottom row

The present study also applied neutrality tests to determine the signatures of natural selection on the *ldh* gene of *P. falciparum*. The significant negative values of Tajima’s *D*, Fu and Li’s D* and Fu and Li’s F* tests implied that the genetic conservation in LDH was due to negative purifying selection. This result was also in agreement with a *d*_*N*_–*d*_*S*_ analysis. These results supported the view that LDH is highly conserved likely due to functional constrains. During the erythrocytic cycle, malaria parasites depend exclusively on anaerobic metabolism for adenosine triphosphate (ATP) production and consequently exhibit high levels of glucose consumption [40–43]. LDH is an enzyme responsible for the conversion of pyruvate to l-lactate while regenerating NAD^+^ from NADH+H^+^ for continued use in glycolysis. The analysis showed that all 7 non-synonymous SNPs that defined 5 isoforms of LDH were located in the regions that are unlikely to cause significant conformation changes, as shown in molecular 3D structure modelling studies of LDH variants in *Plasmodium* species [19, 44, 45]. The analysis also showed that the 9 alleles of *P. falciparum ldh* had identical amino acids at catalytic residues (R95, D155, R158, H182, see Fig. 4), the active site (K84), cofactor binding sites (P235 and P239) and the substrate specific loop (DKEWN, amino acid positions 90–94) [46]. This demonstrates the significance of functional constraints that limited genetic variability of this gene, supporting the view that the *Plasmodium* LDH enzyme should be an attractive target for development of selective inhibitors.

In addition to its role as an anti-malaria drug target, *Plasmodium* LDH is mainly used in screening of clinical malarias in endemic countries. *ldh* genes of *P. falciparum* were cloned and expressed in *Escherichia coli* for productions of polypeptides [46], which were subsequently used in the production of monoclonal antibodies. Different monoclonal antibodies against LDH have been incorporated into RDTs, such as OptiMAL^®^, enabling detection and differentiation of *Plasmodium* species infecting humans. The sensitivity and specificity of each of these tests have been assessed over a range of clinical conditions. In the case of OptiMAL for *P. falciparum* detection, the results of field studies appear more variable with an overall sensitivity between 31.4 and 100%. It has been suggested that the genetic diversity of *P. falciparum ldh* might influence the sensitivity of RDT kits [1–5, 7]. However, global analysis of *P. falciparum LDH* sequences revealed that the allele *L1a* appeared to be the major allele of *LDH*, representing between 80 and 100% of isolates in *P. falciparum* populations of various geographical origins. Thus, it is highly unlikely that the poor sensitivity of the LDH-based detection would be influenced by genetic polymorphism in the *ldh* locus. Additionally, some of this variability may be explained by the relatively poor performance of RDTs at low parasitaemia (> 500 parasites/μl) [7]. Alternatively, it was shown that *P. falciparum* trophozoites had a relatively higher mRNA expression as well as LDH enzyme activity than rings and schizonts, coinciding with the time of maximal metabolic activity by the parasites, and trophozoites were the most susceptible stage of the anti-LDH drugs [47]. Thus, it is suggested that the sensitivity of RDT-tests could be influenced by the stages of erythrocytic development. Whether different erythrocytic stages of *P. falciparum* may account for variability in the sensitivity of LDH-based RDTs will require further investigation.

Currently, LDH peptides representing different regions of *P. falciparum* LDH-1 were chosen for productions of antibodies, which were subsequently incorporated in immunodiagnostic assays [3, 48–51]. The comparative sequence analysis of LDH epitopes and different isoforms of *P. falciparum* LDH indicated that some epitopes, incorporating amino acid residues 3–28, 82–105 and 85–99, contain polymorphic amino acid residues, which differentiate LDH-1 from LDH-3 and LDH-4 (see Additional file 5). Since LDH-3 and -4 isoforms represents < 1% in *P. falciparum* populations (see Table 2), this implies that most commercial immunodiagnostic assays and/or RDTs based on monoclonal antibodies against LDH-1 for *P. falciparum* detection should remain effective in all *P. falciparum* populations. Furthermore, when the LDH epitope sequences were compared with LDH sequences of other human *Plasmodium* species, it was found that LDH epitopes of *P. falciparum* contains a number of polymorphic sites that were different from *P. vivax* LDH. This was consistent with a previous finding that antibodies generated using these epitopes could detect *P. falciparum* with high specificity, but not *P. vivax* [48]. However, the comparative sequence analysis also revealed that some regions of LDH were conserved in all *Plasmodium* species, including KEWNRDDLLPLNNK (amino acid residues 74–87), LKRYITVGGIPLQEF (amino acid residues 172–186), ASPYVAPAAAIIEMAE (amino acid residues 216–231), CSTLLEGQYGH (amino acid residues 243–253). Thus, these sequences may be selected for productions of pan-malarial antibodies for detection of all *Plasmodium* species.

Wright’s fixation index (*F*_*st*_) indicated that allelic distribution patterns of *P. falciparum ldh* in South America were similar to those in Africa and Asia. This result was mainly because all 3 parasite populations shared the same allele of *ldh*, *L1a*. This finding may suggest the evidence of gene flow between the parasite populations on different continents. This finding was supported by a microsatellite and SNP study that addressed the multiple introductions of African *P. falciparum* to South America [52]. The *F*_*st*_ analysis also indicated that *ldh* patterns in Africa and Asia were genetically distinct. This finding suggested that novel *ldh* alleles of *P. falciparum* in Africa and Asia may have arisen independently. The data showed that LDH-2 was more common in Africa, while it was extremely rare in *P. falciparum* in Asia. Further investigation will be required to determine whether LDH-2 has selective advantages over other isoforms.

Finally, sequence analysis of *ldh* from different species of human-derived *Plasmodium* revealed different levels of genetic diversity. The data indicated that the nucleotide sequence diversity of *ldh* in *P. falciparum* was lower than that of *P. ovale* and *P. vivax*. The present study was in general agreement with the recent reports of comparative genomic analysis showing more polymorphism in the *P. vivax* genome than in *P. falciparum* [53]. The results also revealed that the genetic diversity *P. falciparum ldh* was relatively lower than that of simian parasites. The paucity of genetic polymorphism in *P. falciparum ldh* may be indicative of a recent bottle neck and is analogous to the hypothesis by Rich stating that the origin of extant global populations of *P. falciparum* that have recently evolved from a single ancestral population [54].

The genealogy of *Plasmodium* species inferred from the phylogenetic tree constructed using *ldh* sequence revealed *ldh* sequences clustered according to the species of the malaria parasites. An exception was that *ldh* sequences of *P. falciparum* and *P. praefalciparum* were clustered in the same clade, thereby confirming the close evolutionary proximity between the two species. This finding was consistent with mitochondrial and genomic DNA studies, which have demonstrated the shared common origin of these parasites and supported the view that *P. praefalciparum* was the most recent common ancestor of *P. falciparum* [20, 21]. This result was because all *P. praefalciparum ldh* sequences (excluding HM235127 and HM235119) had sequences identical to the *L1a* allele of *P. falciparum*. The tree also indicated that the human malaria parasite *P. falciparum* was more evolutionary related to the malaria parasite in gorillas and chimpanzees than any other species of human malarias. It should be noted that *P. ovale* and *P. malariae*, which were clustered with *P. vivax* according to the phylogenetic tree based on the mitochondrial DNA sequences [55], were not monophyletic with *P. vivax* in *ldh*-based trees. Instead, *P. vivax ldh* formed a monophyletic clade with the malaria parasites of macaques, supporting the evolutionary closeness of *P. vivax*, *P. cynomolgi* and *P. knowlesi*.

## Conclusion

This study extended the understanding of genetic variation in *ldh* and the prevalence of *ldh* alleles in natural populations of *P. falciparum* in Thailand and other endemic areas worldwide. *ldh* sequences of *P. falciparum* in Thailand are mono-allelic for the *L1a* allele. The *L1a* allele was also the major allele of *ldh* in *P. falciparum* in Asia, Africa and South America, implying that the contribution of genetic diversity of *P. falciparum ldh* to the poor sensitivity of RDT is highly unlikely. The genetic conservation of *ldh* in *P. falciparum* makes this gene an excellent target for anti-malaria drug development as well as a key target for RDT detection. Sequence information of *ldh* also recovered a close evolutionary relationship of the human malaria parasite *P. falciparum* and parasites of the African great apes.

## Additional files

**Additional file 1.** Nucleotide sequence IDs of the *ldh* gene of *Plasmodium falciparum* in Thailand.

**Additional file 2.** Nucleotide sequence IDs of the *ldh* gene of *Plasmodium falciparum* from different geographical locations.

**Additional file 3.** Nucleotide sequence IDs of the *ldh* gene of the malaria parasites of humans and non-human primates.

**Additional file 4.** Neighbour Joining tree of 61 allelic sequences of the gene encoding lactate dehydrogenase (*ldh*) from 12 *Plasmodium* parasite species. The sequences are named according to parasite species and allelic type. The first two letters indicate parasite species: *Pf* (*Plasmodium falciparum*), *Pm* (*Plasmodium malariae*), *Po* (*Plasmodium ovale*), *Pv* (*Plasmodium vivax*), *Pp* (*Plasmodium praefalciparum*), *Pr* (*Plasmodium reichenowi*), *Pbi* (*Plasmodium billcollinsi*), *Pbl* (*Plasmodium blacklocki*), *Pa* (*Plasmodium alderi*), *Pg* (*Plasmodium gaboni*), *Pk* (*Plasmodium knowlesi*) and *Pc* (*Plasmodium cynomolgi*). Species showed on the right hand site are labelled with color representing parasite host: *Homo sapiens* (blue), *Gorilla gorilla* (black), *Pan troglodytes* (green) and *Macaca fascicularis* (red). The tree was constructed using the aligned sequences of 768 nucleotides, corresponding to nucleotide position 52–819 after *P. falciparum* strain 3D7. Bootstrap values are shown next to the nodes. Scale bar shows nucleotide substitution per site.

**Additional file 5.** Sequence alignment of *Plasmodium falciparum* LDH epitopes in immunodiagnostic assays and LDH sequences of human *Plasmodium* species. Only LDH sequences that were different from LDH epitopes were shown. Letters in grey indicate polymorphic amino acid residues. Dot (.) indicates an amino acid residue identical to that of LDH epitopes.
