# Supplementary material for: Global sequence diversity of the lactate dehydrogenase gene in Plasmodium falciparum
Source: Malar J. 2018 Jan 9;17:16. doi: 10.1186/s12936-017-2157-5 (PMC5761093; doi:10.1186/s12936-017-2157-5)
Supplement: Supplementary file 4 — Additional file 4. Neighbour Joining tree of 61 allelic sequences of the gene encoding lactate dehydrogenase (ldh) from 12 Plasmodium parasite species. The sequences are named according to parasite species and allelic type. The first two letters indicate parasite species: Pf (Plasmodium falciparum), Pm (Plasmodium malariae), Po (Plasmodium ovale), Pv (Plasmodium vivax), Pp (Plasmodium praefalciparum), Pr (Plasmodium reichenowi), Pbi (Plasmodium billcollinsi), Pbl (Plasmodium blacklocki), Pa (Plasmodium alderi), Pg (Plasmodium gaboni), Pk (Plasmodium knowlesi) and Pc (Plasmodium cynomolgi). Species showed on the right hand site are labelled with color representing parasite host: Homo sapiens (blue), Gorilla gorilla (black), Pan troglodytes (green) and Macaca fascicularis (red). The tree was constructed using the aligned sequences of 768 nucleotides, corresponding to nucleotide position 52–819 after P. falciparum strain 3D7. Bootstrap values are shown next to the nodes. Scale bar shows nucleotide substitution per site. [file 12936_2017_2157_MOESM4_ESM.docx]

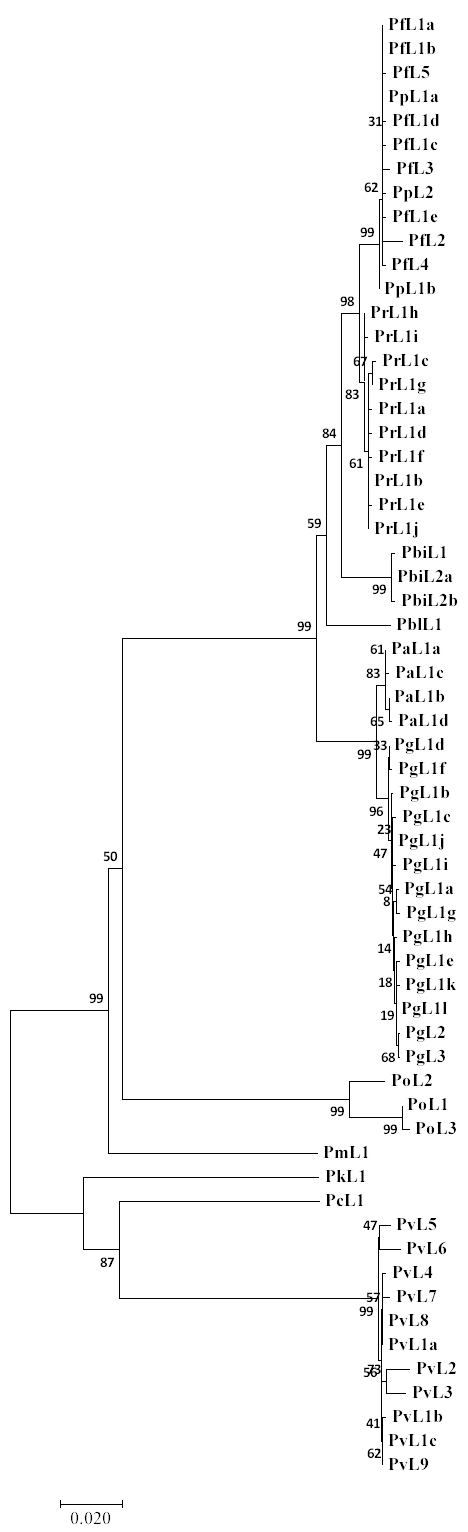


***P. falciparum***

***P. praefalciparum***

***P. reichenowi***

***P. billconlinsi***

***P. blacklocki***

***P. alderi***

***P. gaboni***

***P. ovale***

***P. malariae***

***P. knowlesi***

***P. cynomolgi***

***P. vivax***

**Additional file 4**. Neighbour Joining tree of 61 allelic sequences of the gene encoding lactate dehydrogenase (LDH) from 12 *Plasmodium* parasite species. The sequences are named according to parasite species and allelic type. The first two letters indicate parasite species: ***Pf*** (*P. falciparum*), ***Pm*** (*P. malariae*), ***Po*** (*P. ovale*), ***Pv*** (*P. vivax*), ***Pp*** (*P. praefalciparum*), ***Pr*** (*P. reichenowi*), ***Pbi*** (*P. billcollinsi*), ***Pbl*** (*P. blacklocki*), ***Pa*** (*P. alderi*), ***Pg*** (*P. gaboni*), ***Pk*** (*P. knowlesi*) and ***Pc*** (*P. cynomolgi*). Species showed on the right hand site are labelled with color representing parasite host: *Homo sapiens* (blue), *Gorilla gorilla* (black), *Pan troglodytes* (green) and *Macaca fascicularis* (red). The tree was constructed using the aligned sequences of 768 nucleotides, corresponding to nucleotide position 52-819 after *P. falciparum* strain 3D7. Bootstrap values are shown next to the nodes. Scale bar shows nucleotide substitution per site.
